# A Repeat Random Survey of the Prevalence of Falsified and Substandard Antimalarials in the Lao PDR: A Change for the Better

**DOI:** 10.4269/ajtmh.15-0057

**Published:** 2015-06-03

**Authors:** Patricia Tabernero, Mayfong Mayxay, María Julia Culzoni, Prabha Dwivedi, Isabel Swamidoss, Elizabeth Louise Allan, Maniphone Khanthavong, Chindaphone Phonlavong, Chantala Vilayhong, Sengchanh Yeuchaixiong, Chanvilay Sichanh, Sivong Sengaloundeth, Harparkash Kaur, Facundo M. Fernández, Michael D. Green, Paul N. Newton

**Affiliations:** Lao-Oxford-Mahosot Hospital-Wellcome Trust Research Unit (LOMWRU), Microbiology Laboratory, Mahosot Hospital, Vientiane, Lao PDR; Worldwide Antimalarial Resistance Network (WWARN), Nuffield Department of Clinical Medicine, University of Oxford, Oxford, United Kingdom; Centre for Tropical Medicine and Global Health, Nuffield Department of Clinical Medicine, University of Oxford, Oxford, United Kingdom; Faculty of Postgraduate Studies, University of Health Sciences, Vientiane, Lao PDR; School of Chemistry and Biochemistry, Georgia Institute of Technology, Atlanta, Georgia; Facultad de Bioquímica y Ciencias Biológicas, Universidad Nacional del Litoral-CONICET, Ciudad Universitaria, Santa Fe, Argentina; Division of Parasitic Diseases and Malaria, U.S. Centers for Disease Control and Prevention (CDC), Atlanta, Georgia; London School of Hygiene and Tropical Medicine (LSHTM), London, United Kingdom; Centre of Malariology, Parasitology and Entomology (CMPE), Lao PDR; Bureau of Food and Drug Inspection (BFDI), Ministry of Health, Government of the Lao PDR; Food and Drug Department (FDD), Ministry of Health, Government of the Lao PDR

## Abstract

In 2003, a stratified random sample survey was conducted in the Lao People's Democratic Republic (Laos) to study the availability and quality of antimalarials in the private sector. In 2012, this survey was repeated to allow a statistically valid analysis of change through time. The counterfeit detection device 3 (CD-3) was used to assess packaging quality in the field and HPLC and mass spectroscopy analysis chemical analysis performed. The availability of oral artesunate monotherapies had significantly decreased from 22.9% (22) of 96 outlets in southern Laos in 2003 to 4.8% (7) of 144 outlets in 2012 (*P* < 0.0001). All the samples collected in the 2012 survey contained the correct active pharmaceutical ingredients (APIs) in contrast to the 21 (84%) falsified artesunate samples found in the 2003 survey. Although none of the medicines found in 2012 survey had evidence for falsification, 25.4% (37) of the samples were outside the 90–110% pharmacopeial limits of the label claim, suggesting that they were substandard or degraded. Results obtained from this survey show that patients are still exposed to poorly manufactured drugs or to ineffective medicines such as chloroquine. The quality of artemisinin-based combination therapies (ACTs) used in Laos needs to be monitored, since falsified ACTs would have devastating consequences in public health.

## Background

There is increasing awareness that poor quality medicines, both falsified (counterfeit) and substandard, are important impediments to public health.^1^–^5^ The consequences of patients taking poor quality medicines range from prolonged sickness to death and societies may lose confidence in otherwise effective medicines and suffer major economic losses. Importantly medicines with low amounts of active pharmaceutical ingredients (APIs) can engender drug resistance of pathogens.^6^,^7^

Artemisinin resistance threatens the efforts to control and eliminate malaria. It has emerged on the Thai–Cambodia border with recent evidence that it has spread to southern Lao People's Democratic Republic (Laos).^7^–^9^ To contain artemisinin resistance and protect artemisinin-based combination therapies (ACTs), World Health Organization (WHO) developed the Global Plan for Artemisinin Resistance Containment (GPARC) in 2011.^10^ Improved access to affordable, quality-assured malaria diagnostics and treatment was one of the main objectives, with special focus on the removal of oral artemisinin-based monotherapies and substandard and counterfeit medicines from the market.^11^

Artemisinin derivatives have been widely available in Laos as oral monotherapies since the mid-1990s but the national policy for treatment of confirmed uncomplicated *Plasmodium falciparum* malaria has been co-formulated artemether–lumefantrine (Coartem^™^) since 2005.^12^ Coartem (Novartis, Suffern, NY) provided by the Global Fund to fight AIDS, Tuberculosis and Malaria was introduced into Laos after demonstration of in vivo malaria drug resistance to chloroquine and sulphadoxine–pyrimethamine (SP).^13^,^14^ ACTs have been free in Laos for all ages in the public sector since 2005, and artemisinin-based monotherapies have been withdrawn since 2008, except artesunate injectables that are, as recommended by WHO, the optimal treatment of severe malaria. Although malaria transmission has been decreasing in northern and central Laos, it is still an important health problem in remote and forested areas inhabited by ethnic minorities and migrant workers in the south.^15^,^16^ Given that 6% patients had prolonged *P. falciparum* clearance after oral artesunate in southern Laos, along with the demonstration of the *P. falciparum* Kelch13 mutations,^9^ artemether–lumefantrine is at risk of losing its efficacy in the region.

Reports in the late 1990s in the Mekong region showed high frequencies of poor quality antimalarials,^17^–^20^ with up to 54% of the oral artesunate sample being falsified, usually containing no antimalarial API.^17^,^21^–^26^ However, very few data were acquired using random sampling that would allow objective estimates, with confidence intervals (CIs), of the proportion of a national medicine supply that is substandard or falsified.^5^,^27^ In 2003, in a stratified random sample of the quality of oral artesunate in Laos^28^, “mystery” shoppers were requested to buy artesunate tablets from 180 private sector outlets in 12 (of 18) randomly selected Lao provinces. Of the 25 outlets (13.9%) selling oral artesunate, 22 (88%; 95% CI: 68–97%) sold falsified artesunate, as defined by packaging defects and confirmed by chemical analysis.

In 2012, this survey was repeated, using a similar sampling design to allow, for the first time anywhere, an objective and statistically valid analysis of change over time of the availability and quality of antimalarials in the private sector. Furthermore, it was aimed at examining the change in frequency that outlets sold oral artesunate monotherapy that is no longer recommended, whether genuine, substandard, or falsified. The same methodology that was used in 2003 was followed as much as possible. The main difference was that the 2012 survey was restricted to the five southern Lao provinces as malaria incidence had declined elsewhere in Laos in the intervening 9 years.^16^

## Methods

### Setting.

Laos has a population of ∼6.6 million people with the majority (63%) living in rural areas.^12^,^14^ Malaria transmission has declined over much of the country over the last decade since the introduction of insecticide-treated nets (ITNs)/long-lasting nets (LLNs), and the ACT/rapid diagnostic tests (RDTs),^14^ but there remains a high incidence in the south, especially Savannakhet, Salavan, Sekong, Champasak, and Attapeu Provinces. Ethnic minority groups, forest fringe inhabitants, seasonal migrants, and new forest settlers are especially affected. All age groups are at risk but the majority of patients are adult males and children < 15 years.^12^–^14^,^29^ *P. falciparum* accounts for the majority of *Plasmodium* species (∼87%) with *P. vivax* accounting for ∼13% of infections.

### Study design.

**Figure 1.**
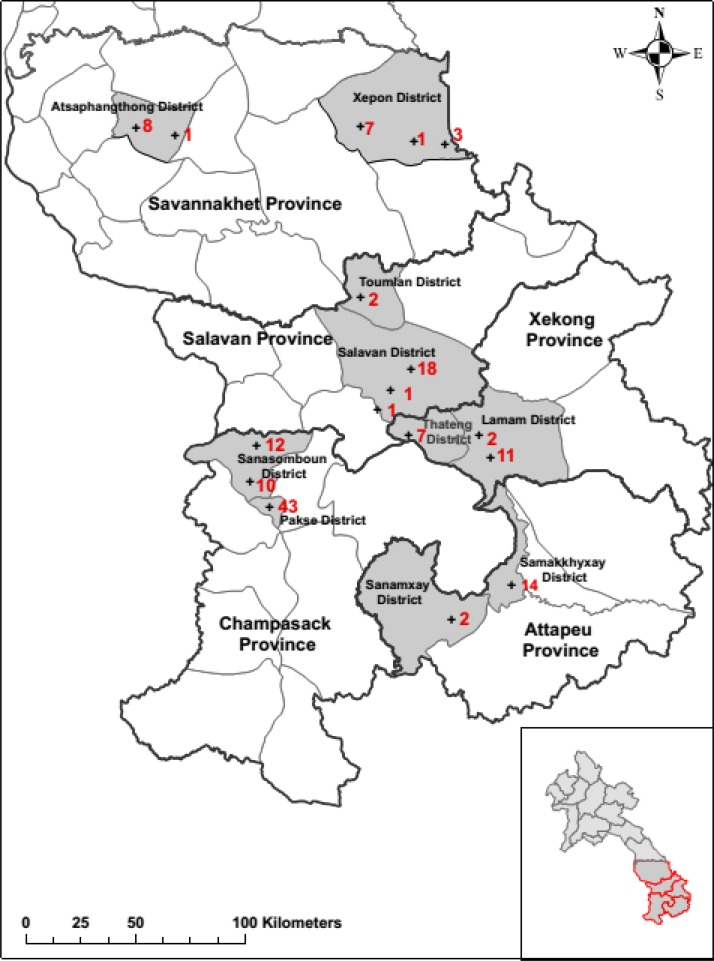
Map of the districts selected and the outlets sampled during the 2012 survey in southern Laos. The (red) numbers refer to the number of outlets sampled within each district.

A cross-sectional random sampling of private medicine outlets was conducted in the five southern Lao provinces starting in September 2012, for 4 weeks. One urban and one rural district (i.e., stratified by urbanization) were selected by random number tables from each of the five southern provinces, and all outlets in these districts were sampled. Districts were categorized by urbanization on the basis of a consensus of the Lao Food and Drug Department (FDD) and Food and Drug Quality Control Center (FDQCC) staff, bearing in mind district population density, transport conditions, and development.^28^ The districts selected were Adsaphangthong and Sepon in Savannakhet, Salavan and Toumlane in Salavan Province, Sekong (Lamarm) and Thateng in Sekong Province, Samakkhixay and Sanamxay in Attapeu, and Pakse and Sanasoumboun in Champasak Province (Figure 1

and Supplemental Table 1).

In the study of Sengaloundeth and others,^28^ only the private sector was sampled. The public government sector is now supplied with quality-assured ACTs.

A 1 week pre-survey training was conducted in Vientiane, Laos, including pretesting of the data collection tools and debriefing process. The questionnaire was translated into Lao and back-translated into English. Training for packaging analysis with the U.S. Food and Drug Administration (FDA) counterfeit detection device 3 (CD-3) was conducted in July 2012.^30^

On arrival in the districts, the study team met with the district and village authorities to update lists of existing licensed pharmacies and unlicensed shops selling medicines within the area. Acting as mystery shoppers, one male and one female assistant procured antimalarial medicines independently from all outlets identified in the selected districts. Outlets were visited by a mystery shopper dressed as a Lao manual worker stating: “*I would like to buy some drugs for my friend who is sick – we are traveling and work in construction– may I see which ones you have so I can choose?*” Once some medicines have been dispensed, the mystery shopper enquired about the availability of other brands or products.

Twenty tablets of each preparation and five vials of parenteral antimalarial medicines were requested for each sample and the number of units available was collected. Three attempts were made to visit outlets closed at the time of the survey. Each assistant was debriefed after each interaction using a semi-structured questionnaire.

Comparative packaging analysis with genuine samples (when available) was performed using the CD-3 later on the same day of collection.

### Inclusion criteria.

All private pharmacy or drug sellers in the study districts, whether registered or unregistered, were eligible for inclusion in the survey. Itinerant drug sellers were not included.

In Laos, private pharmacies are classified depending on the qualifications of the licensee: class I pharmacies are run by a qualified pharmacist with a university degree; class II are run by an assistant pharmacist; class III are run by any medical professional, usually an auxiliary nurse or low-level pharmacist (the licensee does not have to be a pharmacist or an assistant pharmacist). Registered Private Clinics are run by medical doctors after government working hours with at least 7 years of clinical experience in the public sector or by doctors who have retired. Retired pharmacists, nurses, or health workers who have worked for the government for at least 5 years can apply to run any of class I, II, or III pharmacies.^17^,^31^

Poor quality medicines were defined as falsified or substandard based on WHO definitions. Falsified medical products are products deliberately or fraudulently mislabeled with respect to identity or source. In contrast, substandard medicines are genuine medicines produced by manufacturers authorized by the national medicine regulatory authority (MRA), which do not meet quality specifications set for them by national standards.^32^

A sample was defined as a group of apparently physically identical dosage units (e.g., tablets or vials) from one brand and one batch obtained at the same time from the same outlet. Samples were kept in a foam box and sent to Vientiane as early as possible (within 3–4 days of collection) for storage at +4°C (±2°C) in a refrigerator.

All data were double-entered in a pre-established EpiData database. Samples were photographed and/or scanned. Data were analyzed using STATA (v11.2, Stata Corp., College Station, TX) and Microsoft Excel.

### Laboratory analysis of the samples.

Antimalarial samples were sent for chemical analysis to the Centers for Disease Control and Prevention (CDC) and the Georgia Institute of Technology, both in Atlanta, GA.

Mass spectroscopy was used to determine as to whether the sample contained the stated, or other API^33^,^34^ and the content measurements of each API was determined using high-performance liquid chromatography (HPLC).^35^ Between three and five dosage units were tested for each sample (when available) and the mean of the percentage API (%API) was obtained. Twenty-two samples were sent to the London School of Hygiene and Tropical Medicine (LSHTM) for HPLC confirmatory analysis as a quality control measure. Samples were kept in foam boxes protected from light, excessive moisture, or dryness. Temperature data loggers were included within shipments to document appropriate temperature in transit.

Samples were classified as meeting the quality requirements if the amount of API in each of the units, as determined from their content uniformity method,^36^–^40^ lay within the range of 75.0–125.0% of the label claim. The number of units within the 85.0–115.0% and the 90.0–110.0% ranges were also specified (see Supplemental Table 2).

Since “yaa chud” are small plastic bags, commonly sold individually in Laos, containing 4–5 tablets and capsules without any labeling or written instructions,^41^ they were analyzed assuming that they were chloroquine and using the standard dose of 250 mg chloroquine base per tablet. The APIs in all the tablets were confirmed by ultraviolet (UV) spectrum. Results were expressed as percentage of stated or assumed API.

Packaging analysis was conducted, blinded to chemistry and vice versa, by visual inspection and using the U.S. FDA CD-3.^30^ The CD-3 is a nondestructive handheld battery-powered tool that uses multiple light sources to screen for differences in reflectance from the surfaces of dosage forms and their packaging. Suspect medicines are screened in comparison to an authentic product and the differences in appearance are observed. Before September 2012, attempts were made to obtain genuine reference samples of all the different brands and dosage forms of the medicines expected to be sampled in the field. These samples were first screened with the CD-3 to create a library of genuine characteristics and to detect covert package markers for reference.

This report has been written following as far as possible the Medicine Quality Assessment Reporting Guidelines (MEDQUARG)^27^,^42^,^43^ (see Supplemental Table 3). The results have been reported to the Lao FDD and included in the Worldwide Antimalarial Resistance Network (WWARN) Antimalarial Quality (AQ) Surveyor.^5^,^44^ Ethical clearance was granted by the Lao PDR National Ethics Committee for Health Research (NECHR).

## Results

### Survey description.

A total of 147 outlets were sampled in the 10 districts, 45 in the rural and 102 in the urban districts. Of the outlets visited, three outlets were closed during all the three visits and therefore only 144 were included in the study (Supplemental Figure 1).

Registered outlets accounted for 97.2% (140) of those eligible. Pharmacy classes I and II accounted for 30.9% (43) and 30.9% (43) of outlets, respectively, and 33.8% (47) were pharmacy class III. Only 4.1% (6) of the registered outlets were clinics, and one of the outlets sampled was a shop of a registered pharmaceutical manufacturer, Pharmaceutical Factory No. 2. Only four unregistered outlets (2.7%) were found, and they were general shops that were also selling medicines, including antimalarial medication. Two of these shops were in Sepon District, Savannakhet Province, and the other two were in Sanasoumboun District, Champasak Province.

Mystery shoppers were asked by providers if they had a blood test for malaria prior to buying antimalarial medicines in 20.8% (30) of outlets. In 90% (27) of these outlets, the providers suggested that the shopper should have a test for malaria or offered the test himself/herself before buying the medicines. Antimalarials were bought from 76.4% (110) of the eligible outlets. Of the 23.6% (34) outlets where mystery shoppers were unable to buy malaria medicines, in 14.7% (5) of them, the provider refused to sell the drugs without a test even if they had malaria medicines in stock, and 73.5% (25) outlets did not stock antimalarial medicines at the time of the survey. The provider was absent or offered a medicine that was not an antimalarial in four outlets.

### Medicines offered to mystery shoppers at registered and unregistered outlets.

Out of the 158 samples sold for the treatment of malaria, 89.9% (142) were labeled as antimalarials and 10.1% (16) samples were sold as loose tablets with no label or packaging information; nine of these samples were a combination of tablets or capsules with more than one type of medicine (defined as yaa chud as given in reference^41^); the other seven were loose tablets of only one type of medicine sold in plastic bags.

Chloroquine was the medicine most frequently sold, accounting for 79.0% (128) of samples, including in four yaa chud. The other antimalarials sampled were artesunate monotherapy 6.8% (11), quinine 3.7% (6), and artemether–lumefantrine 0.6% (1) (see Table 1).

Tablets accounted for 84.6% (137) of samples, with injections and syrups accounting for 10.5% (17) and 2.5% (4), respectively, including artesunate, quinine, and chloroquine vials.

Nine outlets (6.3%) sold artemisinin derivative monotherapy and a total of eleven samples of artesunate were collected. Seven 50-mg artesunate tablet blister packs were collected (152 tablets) in one outlet in Sepon, one in Salavan, one in Samakkhixay and four outlets in Pakse districts. Six samples were labeled as “Artesunat 50 mg tablets” with Pharbaco Central Pharmaceuticals, Hanoi, Vietnam, as the stated manufacturer and 56% (72) of tablets were labeled as from the same batch. The other brand collected (24 tablets) was “Artesunat 50 mg” with the stated manufacturer as Mekophar Chemical Pharmaceutical Joint, Ho Chi Minh City, Vietnam. No oral artesunate labeled as manufactured by Guilin Pharmaceutical Co. Ltd. (Guilin, PR China) was found.† †Guilin Pharmaceutical Co. Ltd. stopped manufacturing monotherapy artesunate in 2008.

Four artesunate injection samples were sold in two outlets in Sepon and one in Salavan. All the samples were labeled as manufactured by Pharbaco Central Pharmaceuticals.

Three of four bottles of chloroquine 85 mg/mL syrup sold had expired at the time of the survey. One sample of artesunate injection sold, expired a month after the survey ended.

The mean cost of oral chloroquine 250 mg treatment (branded as Maraquine or Malacin) was 632.3 Lao Kips (∼USD 0.07) per tablet; chloroquine injection was 3,428 kip (∼USD 0.42) per vial and chloroquine loose treatment (with no outer packaging) was 361.2 kip (∼USD 0.04) per tablet. The mean cost of artesunate tablet treatment (labeled as Artesunat by Pharbaco Central Pharmaceuticals) was 2,291 kip (∼USD 0.28) per tablet and artesunate injection treatment was 19,500 kip (∼USD 2.40) per vial.

### Chemical quality of the antimalarial medicines.

**Figure 2.**
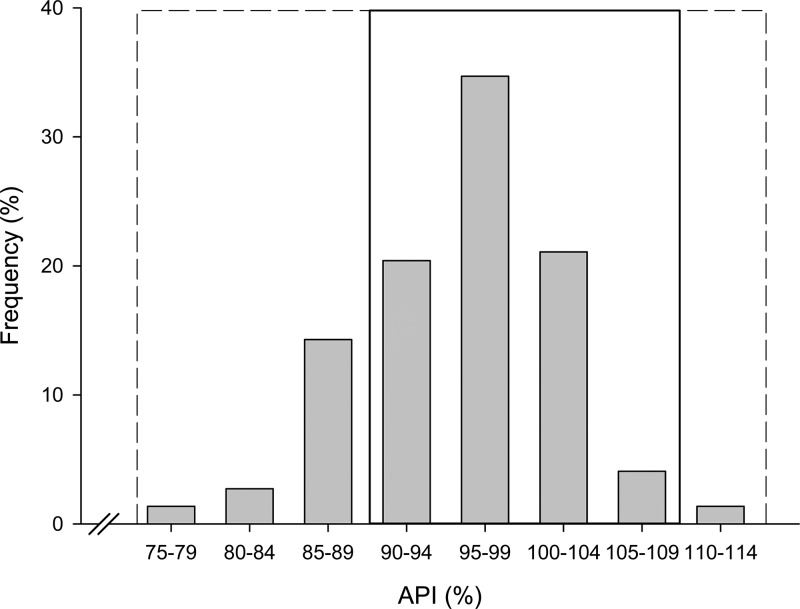
Frequency of antimalarial mean active pharmaceutical ingredient (%API) found in the samples (*N* = 146). Dashed line represents 75–125% cut off and solid line 90–110%.

All of the 146 medicines labeled as antimalarials analyzed (142 samples plus 4 chloroquine tablets found in the yaa chud samples) contained the expected API. All except one sample had all the units with a %API between 75–125% of the content stated on the label. The failed sample was a chloroquine injectable in which 3 of the 4 vials tested had a %API below the 75% cutoff (API values: 64.5%, 70.3%, 62.8%, and 108% per vial). Another nine samples had at least one of their dosage units within a %API range of 75–85%. Seven of these were chloroquine tablets labeled as Maraquine made by CBF Pharmaceuticals, Champasak, Lao PDR; the other two were chloroquine tablets included in the yaa chud (see Figure 2).

The majority of the samples (93.1% [136]) had their unit's %API between 85% and 115%; and 74.6% (109) had their units between 90% and 110%. Twenty-seven (18.5%) samples contained units outside the 90–110% API range but within the 85–115% cutoff; 16.4% (24) samples had units with 85–90% API of the label claim and only 2.0% (3) samples had units > 110% API (see Supplemental Table 4 and 5).

**Figure 3.**
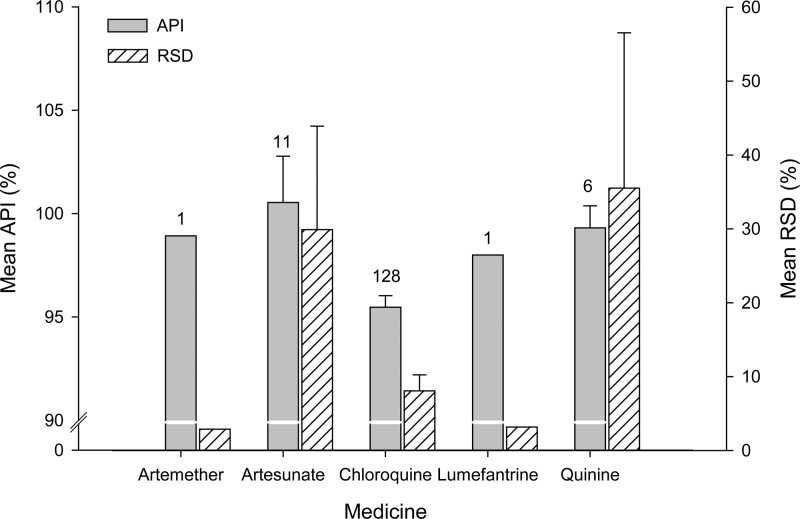
Inter-unit variability measured as the relative standard deviation (RSD) and mean active pharmaceutical ingredient (%API) by medicine. It includes both intravenous and oral forms. Numbers above bars represent sample size.

Inter-tablet variability was also measured for 3–5 tablets per dosage units from the same sample. Artesunate and quinine had high variability between their units with mean (RSD) of 29.9% (95% CI: −1.2; 61.1) and 35.5% (95% CI: −18.5; 89.6), respectively (see Figure 3

, Supplemental Figures 2–4 and Supplemental Table 6).

Results obtained from the confirmatory analysis of 15 samples at the LSHTM were consistent with the analysis in Atlanta with a variation of ±5% between analyses. Higher discrepancies were observed in 6 (4.1% of all) samples with a unit variation ranging from 12.1% to 42.1%. The discordant samples were three 250 mg chloroquine phosphate tablet samples labeled as Maraquine manufactured by CBF Pharmaceuticals, one sample of chloroquine injection 322.5 mg labeled as manufactured by ANB Laboratories (Bangkok, Thailand), one sample of chloroquine tablets 250 mg found in the yaa chud, and one sample of artesunate 60 mg injection stated as manufactured by Pharbaco Pharmaceuticals.

### Packaging analysis with CD-3.

CD-3 analysis was conducted on only 23 samples as genuine comparators were not available for 64.8% (105) of the medicines sampled. An additional 34 samples of loose chloroquine tablets had no outer packaging. All those chloroquine tablets collected had the same tablet design and were consistent with each other under the CD-3 light but were not consistent when analyzed against the only genuine comparator available. No conclusions could be made without the packaging but it suggests that they were from a different manufacturer, brand, or batch when compared with the genuine product. The difference could be due to the excipient variation between different manufacturers or the coating of the chloroquine samples, or medicines may have been degraded due to poor storage conditions.

Another 10 samples were consistent with their genuine comparators, including one artemether–lumefantrine (Coartem), two oral artesunate samples, four oral chloroquine, and three quinine injection vials.

Thirteen samples were not consistent with the genuine comparator when analyzed with the CD-3, including five oral artesunate blisters, five chloroquine injection vials, and three quinine injection vials. These medicines are under investigation with the stated manufacturer to try to understand the significance of the differences observed (Table 2). Discrepancies may be a result of degraded products; artesunate tablets stored at higher temperatures fluoresce more strongly using the CD-3. All except one of the samples that failed packaging analysis had a %API between 90% and 110%, with one chloroquine sample failing both chemistry (mean %API 65.9%) and packaging analysis. This sample (code 4-1601-02-2) may be falsified or possibly substandard.

### Content of yaa chud and loose tablets.

Nine unlabeled plastic bags of diverse medicines were sold to the mystery shoppers when they asked for malaria treatment. Each yaa chud treatment included a median (range) of 5 (3–10) individual plastic bags with 5–7 tablets per capsules per bag. Mass spectrometric tablet analyses demonstrated that each bag contained vitamin B and vitamin C and analgesics, acetaminophen, or ibuprofen. Chloroquine and the antibiotic/antimalarial tetracycline were included in 4 of the 9 yaa chud, but other antibiotics, such as erythromycin, ampicillin, and chloramphenicol, were also found (Supplemental Table 7). Tablets of the sulfonylurea chlorpropamide and the steroid betamethasone were found once in the plastic bags. No artemisinin derivatives were found in the yaa chud unlabeled samples.

Seven samples were dispensed to mystery shoppers as loose monotherapy tablets in plastic bags; six of these bags were dispensed to be taken together with an antimalarial medicine. Six out of seven of these plastic bags contained vitamins: three samples were B group (B_1_ or B_6_), two samples contained vitamin C, and one contained multivitamins. One sample contained diclofenac, a nonsteroidal anti-inflammatory drug. The antimalarial Coartem was given with a bag of vitamin B_1_ and a bag of multivitamins, two samples of chloroquine were dispensed together with two bags of the vitamin B group, and one additional chloroquine sample with loose vitamin C tablets. One oral artesunate was dispensed with a bag of vitamin C tablets and the one bag dispensed without antimalarials contained diclofenac tablets.

The mean cost of yaa chud treatments was 16,333 Lao kips (≈USD 2.03) or 3,098 Lao kips (≈USD 0.38) per bag. Except at two outlets, advice about how to take the yaa chud was given with specification to be taken twice a day (Supplemental Table 7).

## Discussion

**Figure 4.**
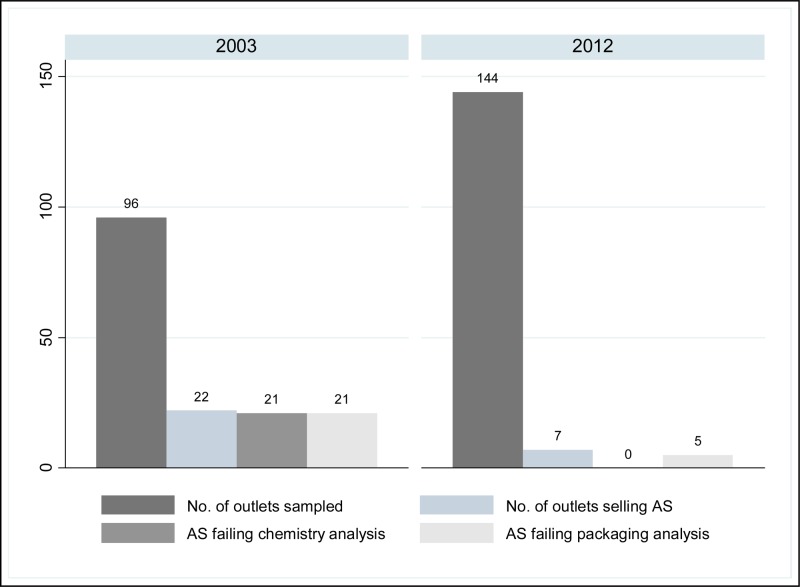
Comparison of availability and quality of artesunate monotherapy tablets samples between the 2003 and 2012 surveys.

Falsified (counterfeit) artesunate was discovered in the late 1990s in mainland southeast (SE) Asia, and contained no or inadequate amounts of artesunate. Concurrent surveys investigating the quality of medicines in Laos demonstrated the availability of poor quality ampicillin, tetracycline, chloroquine, and aspirin ranging from 22% to 46% of 300 and 366 samples, respectively.^17^,^19^,^20^,^45^ The frequency of falsified oral artesunate in the private sector as determined by convenience sampling was as high as 38%.^5^,^21^–^23^,^28^ A random survey in 2003 in Laos estimated that 88% (95% CI: 68–97%) of outlets that sold artesunate, sold falsified artesunate. Results obtained from this 2012 survey in Laos indicate that there has been a significant improvement since 2003, with no falsified artesunate detected (*P* < 0.0001). Importantly, the availability of oral artesunate monotherapies has significantly decreased from 22.9% (22 out of 96 outlets) in southern Laos in 2003 to 4.8% (7 out of 144 outlets) in 2012 (see Figure 4), (non-paired cross-sectional with Fisher's exact test *P* < 0.0001).

Even though the majority of the samples contained the correct mean amount of API, there was a great variation of the quantity of API within the samples and between their units. Although 93.1% samples had all dosage units between 85% and 115% API% of the label claim, nine poor quality samples had dosage units containing between 75% and 85% API. No genuine comparator was available to ascertain whether the samples were falsified.

Of outlet sellers, 14.7% (5) were reluctant to sell malaria medicines without a RDT, and only chloroquine was available in the majority of the outlets. Artemether–lumefantrine Coartem from the public sector was available at only one outlet, in Sepon district, that was part of the Public Private Mix (PPM) program and was therefore not diverted.

Major changes have occurred in the intervening 9 years that may have had a decisive role in the improvement of antimalarial quality in Laos. Control strategies such as ITNs/LLNs, malaria early diagnosis and adequate treatment (EDAT) policy and an increased level of surveillance and reporting through village health volunteers have probably had a positive impact on malaria incidence. Malaria diagnosis and treatment have been decentralized down to the village level with a focus on ethnic minorities in remote areas.^46^ It is likely that patients have better access to antimalarials and health services than they did in 2003.^14^

The dramatic reduction in trade and use of artesunate monotherapy that was available and falsified on an enormous scale 10 years ago is the major driver for the positive change. Guilin Pharmaceuticals Co. Ltd. stopped trading artesunate monotherapy tablets in 2008. In addition, in 2005 Lao started using Global Fund ACTs in the public sector, and in 2008 the PPM program for malaria started,^14^,^46^ presumably reducing the need for patients to seek monotherapy. Six, out of the ten districts sampled, were part of the PPM program.

Pharmaceutical legislation has also been updated and a new quality assurance system has been implemented with technical and financial support from international organizations. The Law on Drugs and Medical Products was amended to ensure adequate registration, distribution, and supply of all medical products. Furthermore, four regional warehouses and one central warehouse in Vientiane were constructed with support from Japan International Cooperation Agency (JICA).^20^,^31^,^46^–^48^ Institutional capacity of the FDQCC in drug analysis has also been improved and pharmacy inspectors trained by the Drug Quality and Information (USP DQI) project from the United States Pharmacopeia (USP) and the WHO.^47^ Since 2003, 12 sentinel sites have been recruited using the Global Pharma-Health Fund (GPHF) Minilab^®^.^45^ Training on good laboratory practice (GLP), good distribution practice (GDP), good procurement practice (GPP), and good manufacturing practice (GMP) has been conducted.

Programs aimed at increasing awareness and communication campaigns against falsified medicines have been conducted, but their efficacy has not been measured.^22^,^49^–^51^ In this survey, out of 144 outlets, 11.8% (17) had some form of information posted on falsified medicines and 4.8% (7) had the WHO “cobra” poster.^52^ One-third (31.6%) of the samples collected had no labeling or no outer packaging. Half of the medicines collected (50%) were labeled in Lao language, 9.5% samples were labeled in Thai, and 6.9% samples were labeled in Vietnamese (all the artesunate samples) with 1.9% in French. It is highly recommended that medicines should be sold in their original packaging and the dose regimes specified in the local Lao language should be included. It is important to note that some artesunate monotherapy was still available, which is not recommended and that some samples collected in the 2012 survey were not registered (the oral artesunate, chloroquine 250 mg tablets labeled as from ANB Laboratories, Cloquine and Nivaquine). All these suggest that further interventions are required.

The significance of content variability^38^ is unclear as there is little objective evidence, informed by pharmacokinetic–pharmacodynamic analysis, as to how many failed dosage units in a sample should be regarded as minimally acceptable; ideally it should clearly be none. There have been reports describing dose-to-dose variations in suspected falsified medicines, and this needs further study.^53^

There is a great difficulty in differentiating substandard and degraded medicines as there is usually insufficient chemical information available to distinguish them. The importance of degradation for the samples collected in this survey is unclear. No outlet stored their medicines in an air conditioned room, 95.8% (138) of 144 were stored in cabinets or drawers inside the shop, and only 4.1% (6) dispensed their medicines from an open shelf or a container.

Despite the apparent improvement in reduction of the availability of oral artesunate monotherapy, the presence and permitted sale of yaa chud was identified as a key issue. Some yaa chud sold to mystery shoppers were found to contain only analgesics that would not have had any antimalarial effect and would have been a waste of patient's money. The finding of steroids and sulfonylureas within yaa chud is also of great concern as they can cause serious side effects. Betamethasone is a glucocorticoid with anti-inflammatory and immunosuppressant effects and has been associated with hypoglycemia and leukocytosis in newborns exposed in utero. Systemic steroids are risk factors for diabetes and hence for melioidosis (*Burkholderia pseudomallei*).^54^ Chlorpropamide, a sulfonylurea for treatment of type 2 diabetes, risks hypoglycemia and is potentially teratogenic.^55^ In addition, 7 (77%) of the yaa chud contained antibiotics as a single dosage unit that lack therapeutic effect and may promote antibiotic resistance. Antibiotics such as chloramphenicol can cause bone marrow toxicity, albeit a rare adverse effect but that would be expected to be fatal in rural Laos. Medical prescription and supervision should be a requisite for dispensing such medicines. Furthermore the high frequency of chloroquine dispensing is alarming as this will not be efficacious against falciparum malaria^11^; vivax malaria accounts for only 5–10% of malaria episodes in Laos. Thus, widespread chloroquine use would inhibit any possible return of falciparum malaria chloroquine susceptibility.^56^ Parenteral chloroquine is no longer recommended for use by WHO^57^ and it should not be available.

The handheld portable CD-3 device was a practical and useful tool with the ability to screen suspicious medicines quickly in the field. However the lack of genuine comparators hampered the number of samples that could be tested, only 35.2%. This emphasizes the need for greater cooperation in obtaining authentic medicines from manufacturers.

The CD-3 analysis picked up some inconsistent samples and variation between batches that are being investigated further. These could have arisen by falsification or by changes of ink, graphics, or pigments during manufacture that we were unaware of. Close collaboration with the pharmaceutical industry will help to clarify such discrepancies (see Supplemental Figure 5).

Although the CD-3 device is portable and very user friendly, sample analysis may be influenced by the subjectivity of the operator. The device will need to be made more robust to be able to empower pharmacy inspectors and customs agents in remote hot and cold monitoring sites or checkpoints in the field.

The study has several important limitations. Given that unlicensed outlets are illegal, it is very difficult to be certain that all such outlets were found and that those found sold the mystery shoppers the medicines that they had in stock. Dissolution and disintegration tests were not performed and the number of dosage units per sample was low. Indeed, a problematic and unresolved issue remains of uncertainty to the number of dosage units that should be collected in the field. Lacking confirmed genuine comparator packets for 105 samples and uncertainty of the diversity of genuine packaging make it difficult to interpret some differences using the CD-3. Furthermore, sample analysis with the CD-3 device may have been influenced by the subjectivity of the operator although, for oral artesunate, diagnostic accuracy was very high.^30^ In both 2012 and 2003 surveys, itinerant sellers were not sampled but they may stock poor quality antimalarials and may reach remote communities.

In SE Asia the private sector is a major source of antimalarial drugs and many patients have relied on artesunate monotherapy in the recent past. As artesunate tablet therapy has not been approved in Laos since 2008, the oral artesunate sold in both surveys was therefore illegal.

Although there are burgeoning reports of an epidemic of falsified ACTs in malarious Africa,^4^ there have been no reports of these in SE Asia. This is encouraging but is inconsistent with recent history and more monitoring of the ACT supply is needed.

## Conclusions

Even though the evidence from these two sequential surveys suggests that the quality of antimalarial medicines has improved over the last decade, the inter-tablet variability found in the samples and the low amounts of API found in some chloroquine vials suggest that substandard medicines may continue to be available in southern Laos. Substandard medicines are important and poorly recognized causes of resistance and poor outcome to individual patients.^6^,^7^ Chloroquine was commonly available raising concern that this antimalarial may be used by private outlets to treat falciparum malaria when it is known to be efficacious only against for *P. vivax*, *P. ovale*, and *P. malariae*. Parenteral chloroquine has no role in antimalarial treatment in Laos and should be removed from circulation. Artesunate oral monotherapy was still available in southern Laos, albeit on a much reduced scale, despite the subsidy on ACTs. Therefore, the strengthening of national and international regulatory oversight is needed.^58^

Despite the apparent improvement in the overall antimalarial quality, the yaa chud pose a serious risk to public health. Patients may be exposed to dangerous drug interactions and adverse effects and to medicines lacking therapeutic efficacy. The low doses of antibiotic found in the yaa chud may also engender drug resistance. Public engagement campaigns, similar to those that have been conducted in Thailand, against yaa chud may help in reducing their use and risks to the population.^41^

Closer collaboration with the pharmaceutical industry will facilitate obtaining authentic medicines necessary to conduct packaging analysis and will help to clarify the observed discrepancies between genuine and suspect samples. Medicines are fundamental to strategies for effective reduction of mortality and morbidity and their good quality is essential for ensuring their efficacy and safety.

## Supplementary Material

Supplemental Datas.

## Figures and Tables

**Table 1:** Description of the antimalarial medicines sampled in the 2012 survey

Labeled as API	No. of samples	%	Dosage (mg)	Form	No. of samples per brand	Stated brand	Stated manufacturer	Mean [price per unit (tab, vial, bottle, bag) 8,064 Lao kips ≈USD 1]
Chloroquine phosphate	85	52.5	250	Tablets	4	Malacin	ANB Laboratories Co., Ltd., Bangkok, Thailand	662.5
			250	Tablets	74	Maraquine	CBF Pharmaceutical Factory Pakse, Champasak, Lao PDR	602.2
			322.5	Injection	7	Malacin	ANB Laboratories Co., Ltd., Bangkok, Thailand	3,428.6
Chloroquine	43	26.5	85	Syrup	4	Chloquine	CBF Pharmaceutical Factory Pakse, Champasak, Lao PDR	12,000
			100	Tablets	3	Nivaquine (chloroquine sulphate)	Sanofi-Aventis, France	750
			Unknown	Tablets	32	Unknown	Unknown	361.2
			Unknown	Tablets	4	Included in yaa chud	Included in yaa chud	Included in yaa chud
Artesunate	11	6.8	50	Tablets	6	Artesunat	Pharbaco Central Pharmaceuticals. Central Pharmaceutical Factory N° 1, Hanoi, Vietnam	2,291.7
			50	Tablets	1	Artesunat	Mekophar chemical pharmaceutical Joint-stock Company, Ho Chi Minh City, Vietnam	1,250
			60	Injection	4	Artesunate	Pharbaco Central Pharmaceuticals. Central Pharmaceutical Factory N° 1, Hanoi, Vietnam	19,500
Quinine dihydrochloride	6	3.7	600	Injection	6	Quinine dihydrochloride	ANB Laboratories Co., Ltd., Bangkok, Thailand	3,291.7
Artemether–lumefantrine	1	0.6	20/120	Tablets	1	Coartem	Novartis Pharmaceuticals Co., Suffern, NY	625
Yaa chud	9	5.5	Unknown	Tablets/capsules	9	Unknown	Unknown	3,098
Loose monotherapy	7	4.3	Unknown	Tablets	7	Unknown	Unknown	517.8

API = active pharmaceutical ingredient; USD = United States dollar.

**Table 2:** Packaging analysis of the samples with the FDA CD-3

API	No of samples collected (*N*)	%	No. of samples with no comparator	No. of samples with no full packaging	CD-3 analysis consistent	
					Yes	No
Artemether–lumefantrine	1	0.6	0	0	1	0
Artesunate	11	6.8	4	0	2	5
Chloroquine	128	79	85	34	4	5
Quinine dihydrochloride	6	3.7	0	0	3	3
Yaa chud/loose tablets	16	9.9	16	0	0	0
Total	162	100	105	34	10	13

API = active pharmaceutical ingredient; CD-3 = counterfeit detection device 3; FDA = Food and Drug Administration;.
