# Supplementary material for: A Repeat Random Survey of the Prevalence of Falsified and Substandard Antimalarials in the Lao PDR: A Change for the Better
Source: Am J Trop Med Hyg. 2015 Jun 3;92(Suppl 6):95–104. doi: 10.4269/ajtmh.15-0057 (PMC4455074; doi:10.4269/ajtmh.15-0057)
Supplement: Supplementary file 1 [file SD5.pdf]

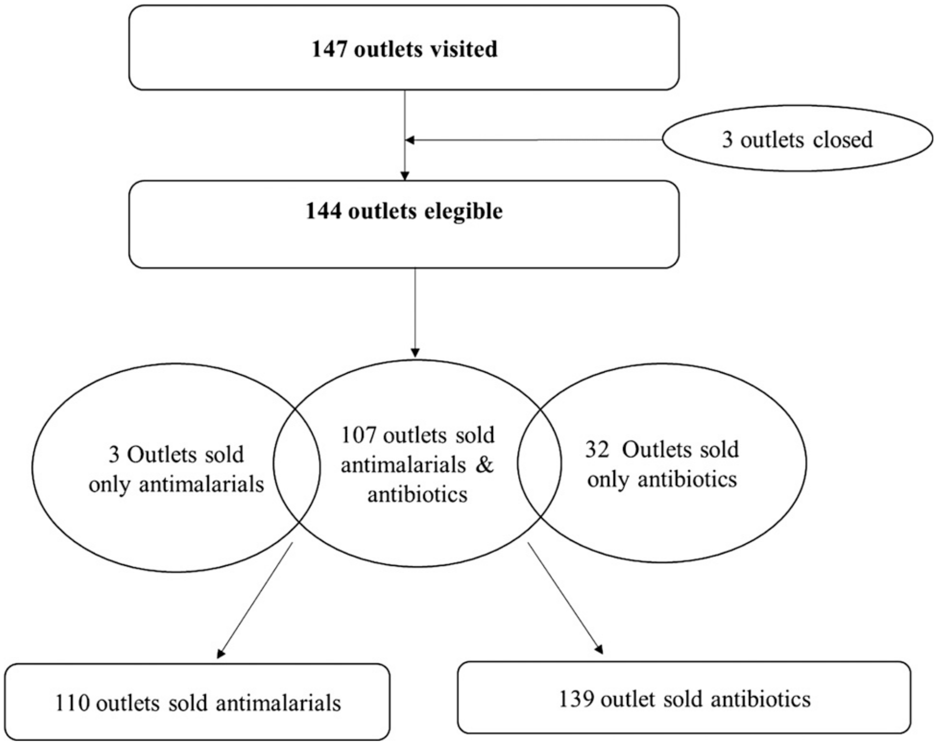

Supplemental Figure 1. Number of outlets included in the antimalarial survey.

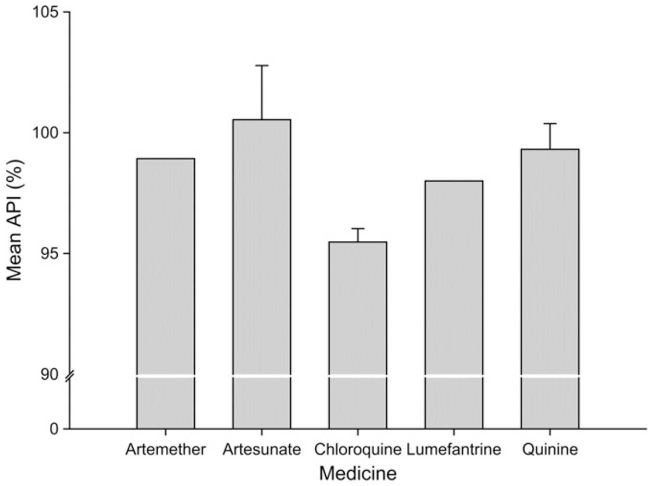

Supplemental Figure 2. Mean %API ± SD of the antimalarial samples collected by medicine. No. of samples: Artemether–lumefantrine N = 1; artesunate N = 11; chloroquine N = 128; quinine N = 6. API = active pharmaceutical ingredient; SD = standard deviation.

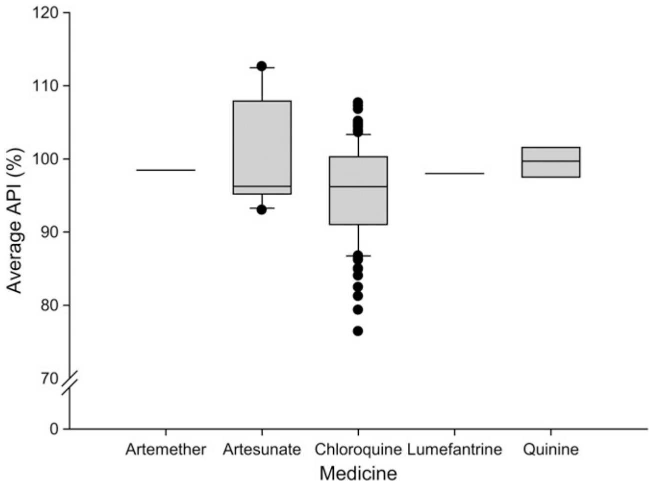

Supplemental Figure 3. Box plot showing the active pharmaceutical ingredient (%API) of the antimalarial samples determined using high-performance liquid chromatography (HPLC) analysis.

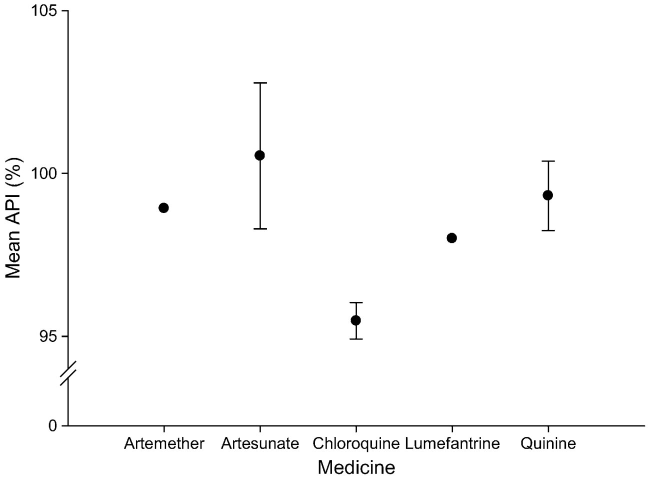

Supplemental Figure 4. Mean API ± SD of the antimalarial samples collected by medicine. API = active pharmaceutical ingredient; SD = standard deviation.

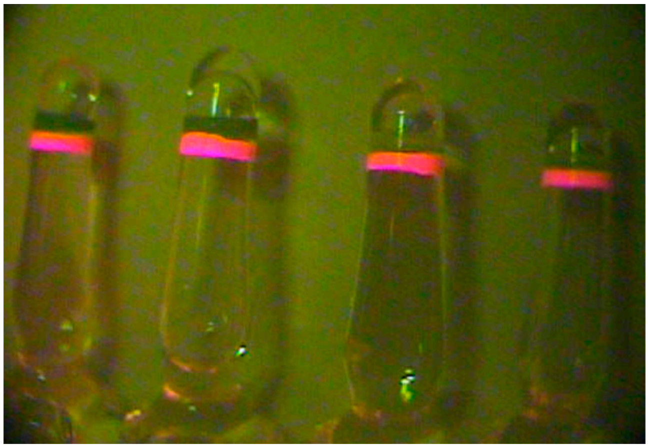

Supplemental Figure 5. Images captured by the CD-3 of genuine Malacin (left two samples) and poor quality Malacin (right two samples) under UV-visible light demonstrating missing green rings on the poor quality sample. CD-3 = counterfeit detection device 3; UV = ultraviolet.

Supplemental Table 1

Random selection of one rural and urban district from each of the five Lao southern provinces

Lao Provinces									
Attapeu		Champasak		Sekong		Salavan		Savannakhet	
Rural	Urban	Rural	Urban	Rural	Urban	Rural	Urban	Rural	Urban
Xanxay	Saysetha	Sanasomboun*	Pakse*	Keleum	Lamarm*	Ta-Oy	Saravane*	Phin	Champhone
Sanamxay*	Samakkhixay*	Bachieng	–	Dakjeung	–	Toumlane*	–	Sepon*	Songkhone
Phouvong	–	Paksong	–	Thateng*	–	Lakhonepheng	–	Nong	Outhoumphone (Seno)
–	–	Pathoumphone	–	–	–	Vapy	–	Thapangthong	Adsaphangthong*
–	–	Phonthong	–	–	–	Khongxedon	–	Xonbouly	Kaysone
–	–	Champasak	–	–	–	Laongam	–	Xaybouly	–
–	–	Soukhoumma	–	–	–	Samouy	–	Vilabouly	–
–	–	Mounlapamok	–	–	–	–	–	Adsaphone	–
–	–	Khong	–	–	–	–	–	Xayphouthong	–
–	–	–	–	–	–	–	–	Phalanxay	–

All outlets in these districts were sampled.
*District selected.

Supplemental Table 2

Cut-off for quality used by the different pharmacopeias for each API

API	Dosage form	United States Pharmacopeia	European Pharmacopeia	International Pharmacopeia (WHO)
Artesunate	Tablets	90.0–110.0%	Not listed	96.0–102.0%
Artesunate	Injectable	95.0–105.0%	Not listed	Not Listed
Artemether–lumefantrine	Tablets	95.0–105.0%	Not listed	97.0–102.0%
Chloroquine hydrochloride	Injectable	47.5–52.5 mg/mL	Not listed	Not listed
Chloroquine phosphate	Tablets	98.0–102.0%	98.5–101.0%	98.0–101.0%
Chloroquine sulphate	Tablets	93.0–107.0%	98.5–101.0%	98.0–101.0%
Quinine sulphate	Tablets	90.0–110.0%	99.0–101.0%	99.0–101.0%
Quinine dihydrochloride	Injectable	Not listed	Not listed	99.0–101.0%
Quinine dihydrochloride	Tablets	Not listed	99.0–101.0%	98.5–101.0%

API = active pharmaceutical ingredient; WHO = World Health Organization.

Supplemental Table 3

MEDQUARG checklist[27] and page number where items are addressed within this manuscript

Section and topic	Item	Description	Page no.
Title/abstract/keywords	1	Identify the article as a study of medicine quality (recommended MeSH headings: "medicine quality, substandard, degraded, counterfeit")	1
		Provide an abstract of what was done and what was found, describing the main survey methods and chemical analysis techniques used	
Introduction	2	Summarize previous relevant drug quality information and describe the drug regulatory environment	3
		State specific objectives	4
Methods			
Survey details	3	The timing and location of the survey; when samples collected and when samples analyzed	4
Definitions	4	The definitions of counterfeit, substandard, and degraded medicines used	6
Outlets	5	The type, including indices of size (e.g., turnover), of drug outlets sampled	7
Sampling design	6	Sampling design and sample size calculation	5 and 6
		Type and number of dosage units purchased/outlet	
		Definition of sampling frame	
		Question of interest, assumptions, sampling method(s) (including method of randomization if random sampling used)	
Samplers	7	Who carried out the sampling and in what guise? What did the collector say in buying the medicines?	5
Statistical methods	8	Describe the data analysis techniques used	6
Ethical issues	9	Whether ethical approval was sought and whether the study encountered any ethical issues	7
Packaging	10	Packaging examination and reference standards	7
Chemical analysis	11	Chemical analysis and dissolution testing SOPs and location(s) of laboratory. Description of validation and reference standards used	6; Reference 33–35; Suppl Table 1
Method validation	12	Details of laboratory method validation results, including but not limited to certificate of analysis for reference standard, within and between run repeatability (RSD% for $N = 5–8$), detection and quantitation limits, accuracy observed for reference samples, linear range for all analytes, sample preparation recovery studies, and selectivity	N/A
		Possibly, validation against a reference method or interlaboratory study	6
Blinding	13	Whether chemistry was performed blinded to packaging and vice versa	7
Results			
Outlets	14	The details of the outlets actually sampled, "class" of pharmacy (e.g., public, private for profit, private not for profit, informal, itinerant)	8
Missing samples	15	The reasons why any outlets chosen for sampling did not furnish a sample. Do these outlets differ systematically from those in which samples were obtained?	N/A
Packaging and chemistry results	16	Packaging and chemistry results and their relationship	9, 10, and 15
		Details of products sampled, how many, in what drug classes, countries of origin, batch numbers, manufacture and expiry dates	8 and 9
		Results for each analysis, packaging, %API, dissolution	9–11
		Additional information could be included in supplementary material	
Category of poor quality medicine	17	A clear statement for each medicine sample detected, whether the investigators class it as genuine, counterfeit, substandard, or degraded with an explanation as to why and whether the medicine was registered with the government in the location(s) sampled	12
State company and address as given on packaging	18	If the names of companies and addresses not given, give a reason as to why this information is not provided	N/A
Sharing data with MRA	19	Whether the data shared with the appropriate MRA and WHO RapidAlert system	7
Dissemination	20	Description of any non-covert packaging features that would allow others to detect counterfeit medicines. If publication is not possible, consider disseminating via web-based supplementary material	N/A
Discussion			
Key results	21	Summarize key results with reference to study objectives	12
Limitations	22	Discussion of limitations of study, especially how robust the estimates of prevalence are and how applicable they may be to wider geographical areas. Discuss the direction and extent of any potential bias	15
Interpretation	23	An interpretation of the results, in conjunction with prior studies, in relation to public health	12
Intervention	24	Whether interventions are thought appropriate and, if so, what type	12–15
Other information			
Conflict of interest	25	State any potential conflicts of interest	16
Funding	26	Give the source of funding and role of funders in the study	17

MeSH = medical subject headings; MRA = medicine regulatory authority; N/A = not applicable; RSD = relative standard deviation; SOPs = standard operating procedures.

Supplemental Table 4

Summary of the chemistry and packaging analysis of the antimalarial samples

API	Dosage (mg)	Form	No. of samples	Stated brand	Stated manufacturer	Chemistry Pass	Chemistry Fail	Packaging Pass	Packaging Fail	%API (average)	HPLC content analysis (%) < 75	75–80	80–85	85–90	90–95	95–100	100–105	105–110	110–115
Chloroquine phosphate	250	Tablets	4	Malacin	ANB Laboratories Co., Bangkok, Thailand	4	0	No comparator		98.83	0	0	0	0	2	0	2	0	0
	250	Tablets	74	Maraquine	CBF Pharmaceutical Factory, Champasak, Lao PDR	74	0	No comparator		92.70	0	0	7	16	23	18	9	1	0
	322.5	Injection	7	Malacin	ANB Laboratories Co., Bangkok, Thailand	6	1	2	5	93.01	1	0	0	0	2	2	2	0	0
Chloroquine	85	Syrup	4	Chloquine	CBF Pharmaceutical Factory, Champasak, Lao PDR	4	0	No comparator		102.25	0	0	0	0	0	2	1	1	0
	100	Tablets	3	Nivaquine (Chloroquine Sulphate)	Sanofi-Aventis, France	3	0	No comparator		102.92	0	0	0	0	0	0	3	0	0
	Unknown	Tablets	36	Unknown	Unknown	36	0	2	No full packaging	95.92	0	1	1	8	4	17	4	1	0
Artesunate	50	Tablets	6	Artesunat	Pharbaco Central Pharmaceuticals, Vietnam	6	0	2	4	95.83	0	0	0	0	2	4	0	0	0
	50	Tablets	1	Artesunat	Mekophar Chemical Pharmaceutical Joint, Vietnam	1	0	0	1	93	0	0	0	0	1	0	0	0	0
	60	Injection	4	Artesunate	Pharbaco Central Pharmaceuticals, Vietnam	4	0	No comparator		109.53	0	0	0	0	0	0	0	1	3
Quinine dihydrochloride	600	Injection	6	Quinine Dihydrochloride	ANB Laboratories Co., Thailand	6	0	3	3	99.26	0	0	0	0	1	3	2	0	0
Artemether–lumefantrine	20/120	Tablets	1	Coartem	Novartis Pharmaceuticals Co., Suffern, NY	1	0	1	0	99.58	0	0	0	0	0	1	0	0	0

API = active pharmaceutical ingredient; HPLC = high-performance liquid chromatography.

Supplemental Table 5

Sample mean %API found by medicine

Medicine	Mean	Mean – SE.	95% CI	*N*
Artemether	98.93	–	–	1
Artesunate	100.54	2.24	95.5–105.5	11
Chloroquine	95.47	0.56	94.4–96.6	128
Lumefantrine	98.00	–	–	1
Quinine	99.31	1.06	96.6–102.1	6

API = active pharmaceutical ingredient; CI = confidence interval; SE = standard error.

Supplemental Table 6

RSD within dosage units of the antimalarial samples

Medicine	RSD – mean	RSD – −95.00%	RSD – +95.00%	*N*
Artemether	2.88	–	–	1
Artesunate	29.90	−1.28	61.09	11
Chloroquine	8.07	3.78	12.36	128
Lumefantrine	3.17	–	–	1
Quinine	35.52	−18.54	89.57	6

RSD = relative standard deviation.

Supplemental Table 7

Description of the Yaa Chud samples bought in the 2012 survey

Yaa chud Code	No of bags given	No. of units per bag	Formulation included in each bag	Medicines included in each bag as determined by mass spectrometry					Cost total treatment in Lao Kip	Cost per bag in Lao Kip	Instructions
				Antibiotic	Analgesic	Vitamins	Antimalarials	Other			
I	3	6	5 tablets + 1 capsule	Tetracycline or doxycycline	Ibuprofen<br>Acetaminophen	Vitamin C<br>Vitamin B_1 and B_6	Chloroquine		10,000	3,333	To be taken once a day
II	10	5	4 tablets + 1 capsule	Tetracycline or doxycycline	Acetaminophen	Vitamin B_6<br>Vitamin B_1	Chloroquine (Maraquine)		30,000	3,000	Take one bag in the morning and one in the evening for 5 days
III	5	6	5 tablets + 1 capsule	Tetracycline or doxycycline	Ibuprofen<br>Acetaminophen	Vitamin C<br>Vitamin B_6	Chloroquine		15,000	3,000	Take one bag in the morning and one bag in the evening
IV	6	6	5 tablets + 1 capsule	–	Acetaminophen<br>Acetaminophen	Vitamin B_1<br>Vitamin B_6<br>Vitamin C		Chlorpropamide	18,000	3,000	Take one bag in the morning and one in the evening for 3 days
V	6	7	7 tablets	Erythromycin	Diclofenac<br>Acetaminophen<br>Acetaminophen<br>Acetaminophen, vitamin B_6 and sugar<br>Acetaminophen	Vitamin C			18,000	3,000	To be taken by adults only
VI	5	7	7 tablets	Ampicillin	Acetaminophen and caffeine<br>Acetaminophen	Vitamin C<br>Vitamin B_6<br>Vitamin B_1		Betamethasone	25,000	5,000	Take one bag in the morning and one bag in the evening
VII	5	5	5 tablets	–	Acetaminophen and Caffeine<br>Acetaminophen<br>Acetylsalicylic acid	Vitamin C<br>Vitamin B_6			15,000	3,000	Not given
VIII	3	5	3 tablets + 2 capsules	Chloramphenicol	Acetaminophen<br>Acetaminophen	Vitamin B_6<br>Vitamin B_6			10,000	3,333	Take one or two per day
IX	6	5	4 tablets + 1 capsule	Tetracycline or doxycycline	Acetaminophen<br>Acetaminophen	Vitamin B_6	Chloroquine		6,000	1,000	Not given
